# Supplementary material for: Structure of the MRAS–SHOC2–PP1C phosphatase complex
Source: Nature. 2022 Jul 13;609(7926):416–23. doi: 10.1038/s41586-022-05086-1 (PMC9452295; doi:10.1038/s41586-022-05086-1)
Supplement: Supplementary file 1 — Raw electrophoretic images. Four display pages with multiple images per page. Uncropped and unprocessed western blot and SDS–PAGE images are presented, with information about the location in the main figures and extended data figures. [file 41586_2022_5086_MOESM1_ESM.pdf]

nature portfolio

https://doi.org/10.1038/s41586-022-05086-1

Supplementary information

Structure of the MRAS–SHOC2–PP1C phosphatase complex

In the format provided by the
authors and unedited

Supplementary Figure 1: related to Figure 4d (Miapaca2 + SHOC2 mutants)

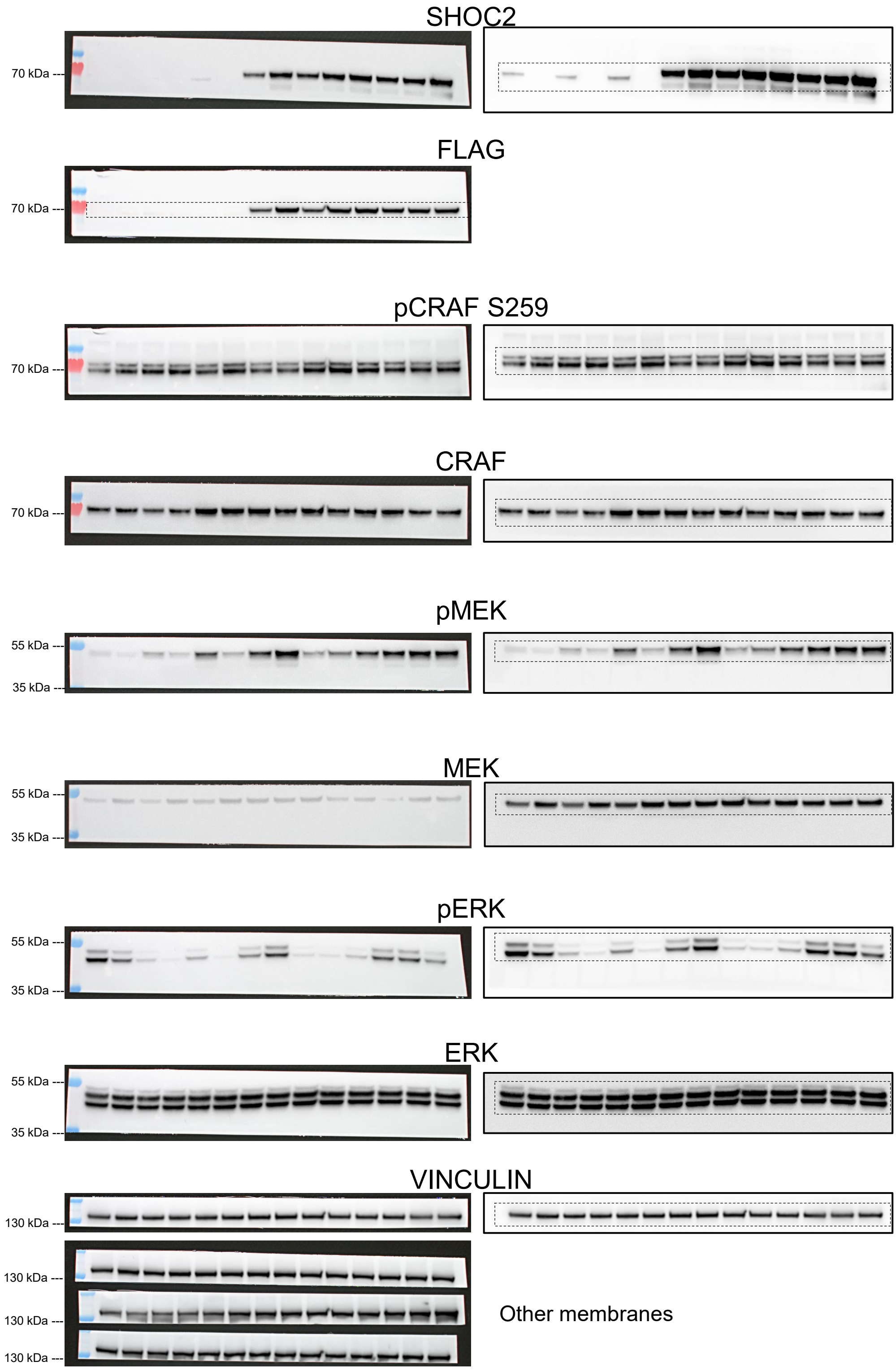

Supplementary Figure 1
Uncropped western blot images used in Figure 4d, with cropping used indicated where appropriate. Membranes were trimmed prior antibody detection and imaging of western blots.

Supplementary Figure 1 continued: Related to Extended Data Figure 7d (MRAS Knockdown with siRNA)

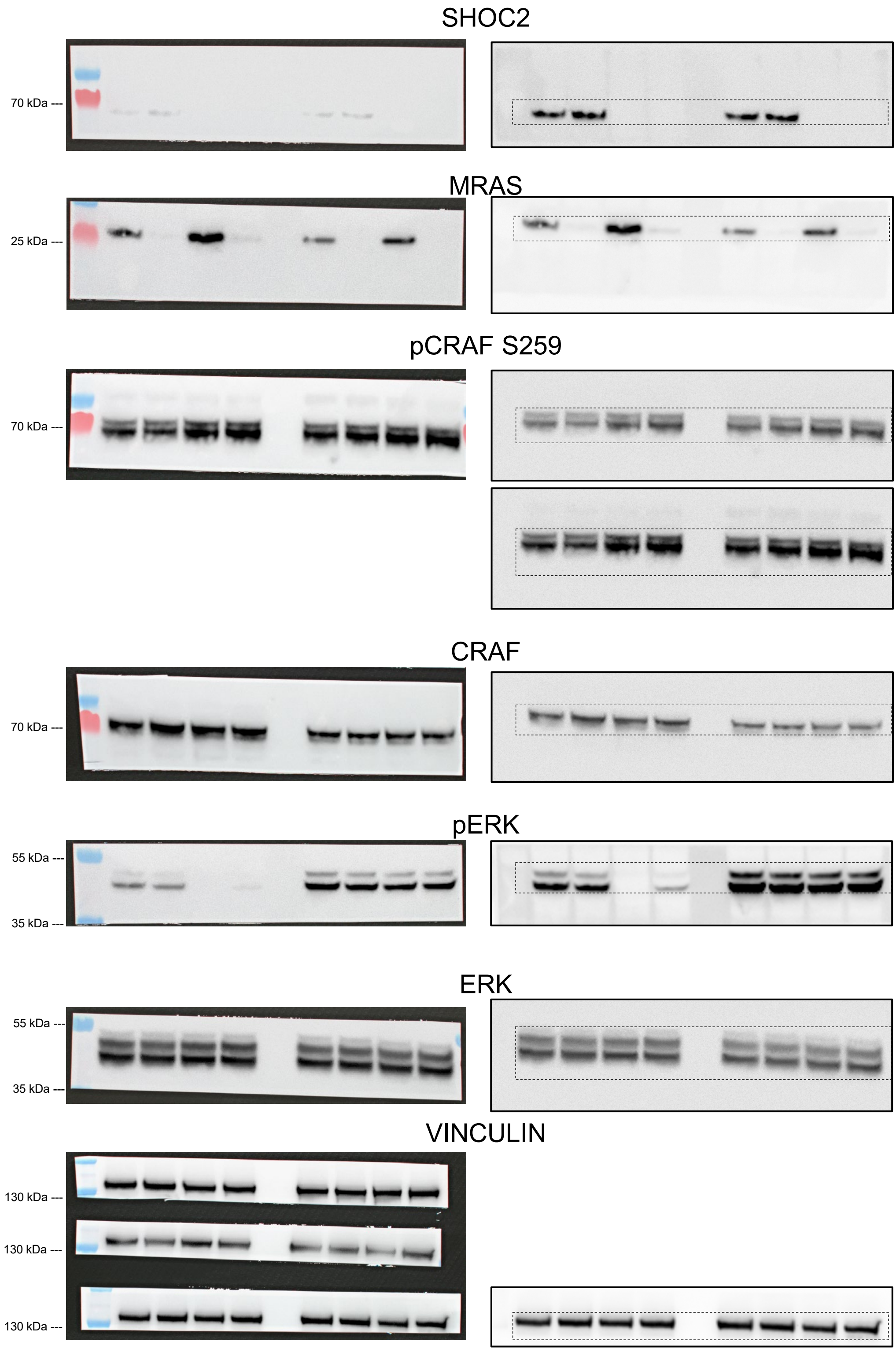

Supplementary Figure 1
Uncropped western blot images used in Extended Data Figure 7d, with cropping used indicated where appropriate. Membranes were trimmed prior to antibody detection and imaging of western blots.

Supplementary Figure 1 continued: Related to Figure 4 (BRAF dephosphorylation assays)

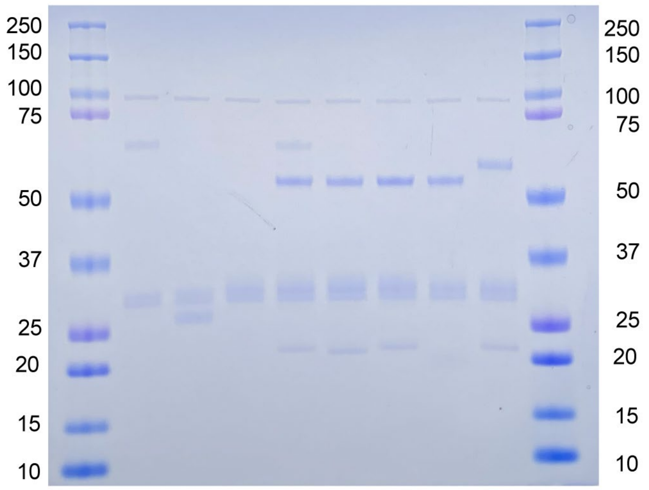

Mekless

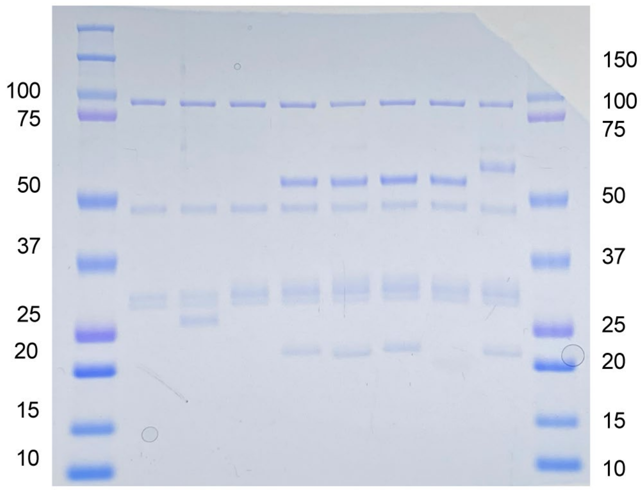

Autoinhibited Complex

Supplementary Figure 1
Uncropped SDS-PAGE gel images used in Figure 4b

Supplementary Figure 1 continued, Related to Figure 4b (BRAF dephosphorylation assays)

pS259(pS365) Blot

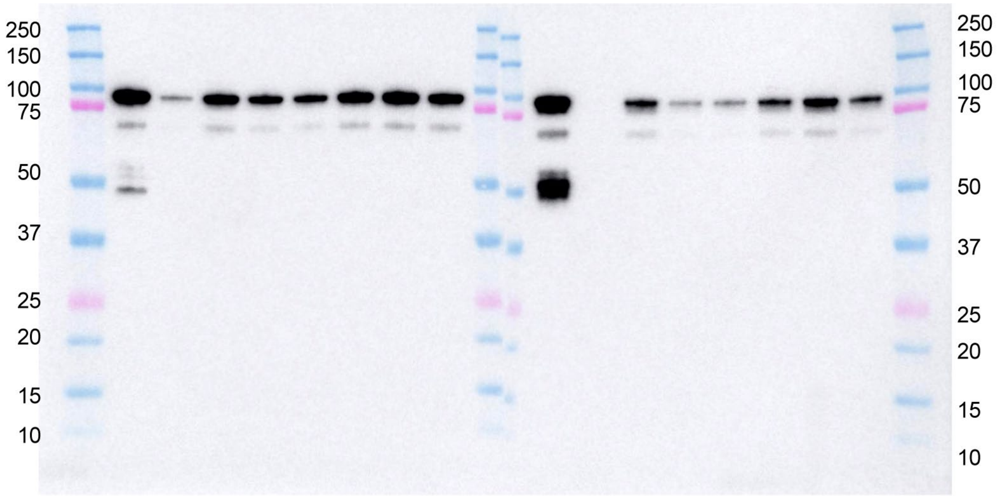

pS729

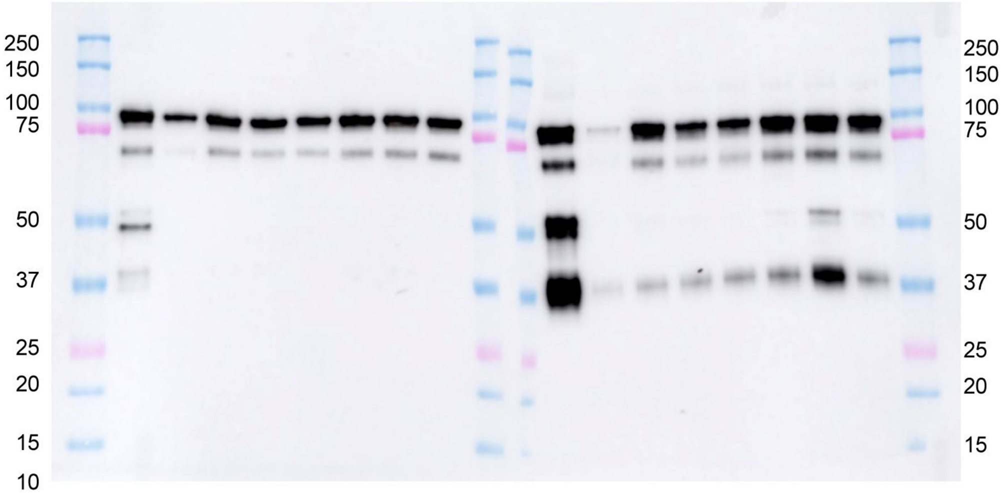

Supplementary Figure 1
Uncropped western blot images used in Figure 4b
