# Supplementary material for: Structure of the MRAS–SHOC2–PP1C phosphatase complex
Source: Nature. 2022 Jul 13;609(7926):416–23. doi: 10.1038/s41586-022-05086-1 (PMC9452295; doi:10.1038/s41586-022-05086-1)
Supplement: Supplementary file 2 — Reporting Summary [file 41586_2022_5086_MOESM2_ESM.pdf]

nature portfolio

Corresponding author(s): Zachary Hauseman, Luca Tordella, Claudio Thoma, Dan King

Last updated by author(s): Jun 13, 2022

# Reporting Summary

Nature Portfolio wishes to improve the reproducibility of the work that we publish. This form provides structure for consistency and transparency in reporting. For further information on Nature Portfolio policies, see our Editorial Policies and the Editorial Policy Checklist.

## Statistics

For all statistical analyses, confirm that the following items are present in the figure legend, table legend, main text, or Methods section.

| n/a | Confirmed | |
|---|---|---|
| ☐ | ☒ | The exact sample size (*n*) for each experimental group/condition, given as a discrete number and unit of measurement |
| ☐ | ☒ | A statement on whether measurements were taken from distinct samples or whether the same sample was measured repeatedly |
| ☒ | ☐ | The statistical test(s) used AND whether they are one- or two-sided<br>*Only common tests should be described solely by name; describe more complex techniques in the Methods section.* |
| ☒ | ☐ | A description of all covariates tested |
| ☒ | ☐ | A description of any assumptions or corrections, such as tests of normality and adjustment for multiple comparisons |
| ☐ | ☒ | A full description of the statistical parameters including central tendency (e.g. means) or other basic estimates (e.g. regression coefficient) AND variation (e.g. standard deviation) or associated estimates of uncertainty (e.g. confidence intervals) |
| ☒ | ☐ | For null hypothesis testing, the test statistic (e.g. *F*, *t*, *r*) with confidence intervals, effect sizes, degrees of freedom and *P* value noted<br>*Give P values as exact values whenever suitable.* |
| ☒ | ☐ | For Bayesian analysis, information on the choice of priors and Markov chain Monte Carlo settings |
| ☒ | ☐ | For hierarchical and complex designs, identification of the appropriate level for tests and full reporting of outcomes |
| ☐ | ☒ | Estimates of effect sizes (e.g. Cohen's *d*, Pearson's *r*), indicating how they were calculated |

*Our web collection on statistics for biologists contains articles on many of the points above.*

## Software and code

Policy information about availability of computer code

Data collection

SEDFIT version 15.01b
Rockmaker Version 3.14.6.6, 3.17.8.1
FUSION FX7 system integrated software
Unicorn 7.1

Data analysis

Gussi
Graphpad Prism 8, 9.2.0
Biacore Evaluation 3.0.12.15655
FloJo V10.7.1
XDS
autoPROC
Phaser 1.18.2_3874+SVN
Coot molecular graphics 0.96 EL
Phenix 1.19.2_4158
Buster 2.11.8
Depmap package v1.8
Experiment hub package V2.2 (Bioconductor)
R 4.1.1
Modelling packages (see methods for references):
3D-RISM
Amber ff19SB

parm@Frosst
AMBER 16
PMEMD CUDA
CPPTRAJ
Getcontacts

For manuscripts utilizing custom algorithms or software that are central to the research but not yet described in published literature, software must be made available to editors and reviewers. We strongly encourage code deposition in a community repository (e.g. GitHub). See the Nature Portfolio guidelines for submitting code & software for further information.

## Data

Policy information about availability of data

All manuscripts must include a data availability statement. This statement should provide the following information, where applicable:

- Accession codes, unique identifiers, or web links for publicly available datasets
- A description of any restrictions on data availability
- For clinical datasets or third party data, please ensure that the statement adheres to our policy

The data supporting the findings of this study are available from the corresponding authors upon reasonable request. Structural factors and coordinates have been deposited in the Protein Data Bank under accession codes 7TYG for the SHOC2 (80-582) apo structure and 7TXH for the SHOC2 M173I (80-582)-MRAS Q71R (1-178)-PP1Ca (7-300) ternary complex structure.

## Human research participants

Policy information about studies involving human research participants and Sex and Gender in Research.

| | |
|---|---|
| Reporting on sex and gender | N/A |
| Population characteristics | N/A |
| Recruitment | N/A |
| Ethics oversight | N/A |

Note that full information on the approval of the study protocol must also be provided in the manuscript.

# Field-specific reporting

Please select the one below that is the best fit for your research. If you are not sure, read the appropriate sections before making your selection.

☒ Life sciences ☐ Behavioural & social sciences ☐ Ecological, evolutionary & environmental sciences

For a reference copy of the document with all sections, see nature.com/documents/nr-reporting-summary-flat.pdf

# Life sciences study design

All studies must disclose on these points even when the disclosure is negative.

| | |
|---|---|
| Sample size | Sample sizes for individual experiments were determined with standard practices in the respective techniques. The number of independent experiments for each type of experiment is noted in the manuscript. Crystallographic data was determined from single crystals for each respective structure. |
| Data exclusions | No data was excluded from this manuscript. For images or experiments that are representative of independent replicates, the number of independent experiments has been noted. |
| Replication | We were able to replicate all data present in the manuscript. |
| Randomization | We did not conduct experiments where randomization was applicable. |
| Blinding | We did not conduct experiments where blinding was applicable. |

# Reporting for specific materials, systems and methods

We require information from authors about some types of materials, experimental systems and methods used in many studies. Here, indicate whether each material, system or method listed is relevant to your study. If you are not sure if a list item applies to your research, read the appropriate section before selecting a response.

## Materials & experimental systems

| n/a | Involved in the study |
|---|---|
| ☐ | ☒ Antibodies |
| ☐ | ☒ Eukaryotic cell lines |
| ☒ | ☐ Palaeontology and archaeology |
| ☒ | ☐ Animals and other organisms |
| ☒ | ☐ Clinical data |
| ☒ | ☐ Dual use research of concern |

## Methods

| n/a | Involved in the study |
|---|---|
| ☒ | ☐ ChIP-seq |
| ☐ | ☒ Flow cytometry |
| ☒ | ☐ MRI-based neuroimaging |

# Antibodies

| | |
|---|---|
| Antibodies used | LANCE Eu-W1024 Anti-6x his, Perkin Elmer # AD0400 / # AD0401 / # AD0402;<br>Anti-BRAF pS365, Cell Signaling Technologies # 921S;<br>Anti BRAF pS729, Abcam # ab124794;<br>Rabbit anti-MRAS, Abcam # ab176570;<br>Mouse anti-Vinculin, Sigma # V9131;<br>Rabbit anti-SHOC2, Cell Signaling Technology # 53600;<br>Rabbit anti-phospho-p44/42 MAPK (Erk1/2) (Thr202/Tyr204), Cell Signaling Technology # 4370;<br>Rabbit anti-p44/42 MAPK, Cell Signaling Technology # 9102;<br>Rabbit anti-phospho-MEK1/2 (Ser217/221), Cell Signaling Technology # 9154;<br>Rabbit anti-MEK1/2, Cell Signaling Technology # 9122;<br>Rabbit anti-phospho-C-Raf (Ser259), Cell Signaling Technology # 9421;<br>Rabbit anti-C-Raf, Cell Signaling Technology # 53745;<br>Rabbit anti-Flag-tag, Cell Signaling Technology # 14793;<br>Anti-mouse IgG HRP linked, Cell Signaling Technology # 7076;<br>Anti-rabbit IgG HRP linked, Cell Signaling Technology # 7074; |
| Validation | Antibodies were not orthogonally validated in-house. Antibodies with as many trusted citations as possible were used. All antibodies functioned as expected for their respective assays. |

# Eukaryotic cell lines

Policy information about cell lines and Sex and Gender in Research

| | |
|---|---|
| Cell line source(s) | HEK293T from ThermoFisher Scientific. MiaPaca2 cells were sourced from ATCC. cells were sourced from SF9 and SF21 cells were from Expression systems. |
| Authentication | Cell lines were not further authenticated. |
| Mycoplasma contamination | HEK293T and MiaPaca2 cells tested negative for mycoplasma contamination. SF9 and SF21 were not tested for contamination. |
| Commonly misidentified lines (See ICLAC register) | No commonly misidentified cell lines were used in this study. |

# Flow Cytometry

## Plots

Confirm that:

☒ The axis labels state the marker and fluorochrome used (e.g. CD4-FITC).

☒ The axis scales are clearly visible. Include numbers along axes only for bottom left plot of group (a 'group' is an analysis of identical markers).

☒ All plots are contour plots with outliers or pseudocolor plots.

☒ A numerical value for number of cells or percentage (with statistics) is provided.

## Methodology

| | |
|---|---|
| Sample preparation | 16k HEK293T cells were transfected with 120ng plasmid in 96 well format 1 day post seeding. 48h after transfection cells were prepared from 96 well standard tissue culture plates for flow cytometry. |
| Instrument | CytoFLEX S (Beckman Coulter) |

| | |
|---|---|
| Software | FloJo V10.7.1 |
| Cell population abundance | Cell sorting was not used in this study. |
| Gating strategy | Gating was based on populations of singlet cells which expressed mCherry signal (shown in extended date figure 6a panel i). To select these populations, transfected cells were compared to untransfected cells to determine minimal mCherry signal for the gate. Please note this is shown in the extended data rather than in Supplementary Information. |

☒ Tick this box to confirm that a figure exemplifying the gating strategy is provided in the Supplementary Information.
